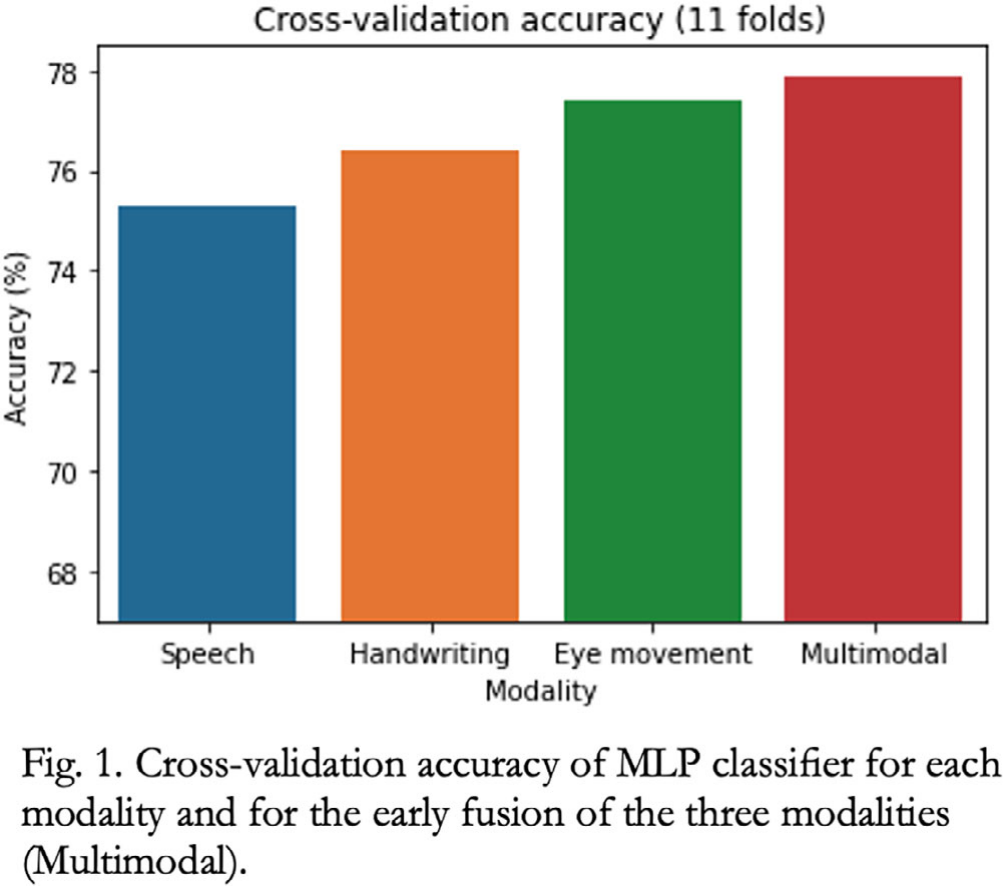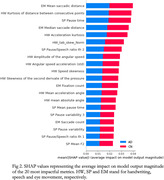# Multimodal characterization of Alzheimer's Disease using speech, eye movement, and handwriting

**DOI:** 10.1002/alz.085472

**Published:** 2025-01-09

**Authors:** Laureano Moro‐Velazquez, Anna Favaro, Thomas Thebaud, Yuzhe Wang, Ankur Butala, Esther S Oh, Najim Dehak

**Affiliations:** ^1^ Johns Hopkins University, Baltimore, MD USA; ^2^ Johns Hopkins University School of Medicine, Baltimore, MD USA

## Abstract

**Background:**

Phenotyping Alzheimer’s Disease (AD) can be crucial to providing personalized treatment. Several studies have analyzed the use of digital biomarkers to characterize a subject’s behavior, usually obtained from a single modality, such as speech. However, combining several modalities in a single study has not been deeply studied. We aim to characterize people with AD and differentiate them from a cognitively normal control group (CN), employing metrics obtained through from three different modalities: speech, handwriting, and eye movement.

**Method:**

We collected data from a group of 11 participants in the early stages of AD or with, mild cognitive impairment due to AD, and an age‐matched CN group of 13 participants. Each participant read out loud the Rainbow Passage, while their speech was recorded with a headset microphone and their eye movement with a high precision eye‐tracker, simultaneously. Then, the participants were asked to draw a spiral on a digital pad. We obtained 57 speech metrics, related to articulation, prosody, and hesitation; 90 handwriting metrics, characterizing drawing entropy, writing speed, and acceleration; 32 eye movement metrics related to saccade, fixation, and blink statistics. Several multilayer perceptron (MLP) classifiers were trained per modality and using all modalities together, following a cross‐validation strategy. SHAP values were calculated to rank metric impact in the differentiation between AD and CN.

**Result:**

Cross‐validation accuracy in the binary classification of AD/CN ranged between 75.3% and 77.4% for single modalities, whereas the combination of the three modalities provided a 77.9% accuracy. The drawing velocity skewness metric provided the highest area under the receiver operating characteristic curve (AUC) from all the handwriting features (0.83), whereas the pause variability was the speech metric with the highest AUC (0.79). The number of fixations provided the best differentiation between groups in the eye movement modality, with AUC=0.69.

**Conclusion:**

Combining the three proposed modalities provides a higher differentiation power than using each modality separately. The combination can provide a more insightful and complementary characterization of patients. Handwriting metrics have the most impact on the prediction of the multimodal MLP model, according to the SHAP values on the multimodal classifier.